# E-learning as valuable caregivers’ support for people with dementia – A systematic review

**DOI:** 10.1186/s12913-019-4641-9

**Published:** 2019-11-01

**Authors:** Blanka Klimova, Martin Valis, Kamil Kuca, Jiri Masopust

**Affiliations:** 1 Department of Applied Linguistics, Faculty of Informatics and Management, University of Hradec Kralove, Rokitanskeho 62, Hradec Kralove, Czech Republic; 2 Department of Neurology, University Hospital Hradec Kralove, Sokolska 581, Hradec Kralove, Czech Republic; 3 Biomedical Research Centre, University Hospital Hradec Kralove, Sokolska 581, Hradec Kralove, Czech Republic; 4 Malaysia Japan International Institute of Technology (MJIIT), Universiti Teknologi Malaysia Kuala Lumpur, Jalan Sultan Yahya Petra, Kuala Lumpur, 54100 Malaysia; 5 Department of Psychiatry, University Hospital Hradec Kralove, Sokolska 581, Hradec Kralove, Czech Republic

**Keywords:** E-learning, Dementia, Caregivers, Benefits, Limitations

## Abstract

**Background:**

Present demographic trends show a considerable rise in elderly populations with aging disorders, such as dementia. The current article focused on the exploitation of e-learning as an informal support for caregivers of people with dementia and considered its benefits and limitations to provide proper and relevant care for this target group of people as well as maintain the quality of life of their caregivers.

**Methods:**

The methodology of this study is based on a literature review of accessible peer-review articles from three recognized databases: Web of Science, Scopus, and PubMed. The findings of the selected studies were compared and evaluated.

**Results:**

The findings showed that e-learning educational programs/courses helped caregivers feel more confident about dementia care, reduced their perceived stress and enhanced their feelings of empathy, understanding and concern.

**Conclusions:**

The findings of this study reveal that the exploitation of e-learning as a support tool, especially for informal caregivers, in the management of dementia may be a promising method, but its implementation requires professional training of informal caregivers in the use of this technology. More evidence-based studies are needed on this topic.

## Background

Dementia affects 58 million people worldwide, and the forecasts indicate that this number will be three times higher by 2050 [1]. One of the foremost and striking symptoms of dementia is cognitive impairment [2], but people with dementia also have problems with concentration, orientation, finding the right words, and planning. They may be prone to rapid mood changes, and they may suffer from depressive symptoms or apathy and exhibit signs of aggression against other people, especially their loved ones [3, 4]. These symptoms generally worsen over time, and people with dementia lose their capability of performing tasks of daily life. Therefore, they are necessarily reliant on the help of other people, in most cases, on their relatives, who become their caregivers [5].

There is a current tendency to shift institutional care to community care [6]. For example, the results of the SHARE project revealed that the non-institutionalized care in Europe of people aged 65 years and over ranged between 21 and 43% [7]. Informal care heavily depends on the help of family members, and the percentage of these informal caregivers has reached 80%. However, the assistance of informal caregivers generally results in a worsening their quality of life [8]. Informal caregivers generally spend between five and 20 h per day taking care of family members with dementia [9]. Informal caregivers, like formal caregivers, also suffer from a gradually increasing physical, mental and economic burden [10–12]. Therefore, there is an urgent need and effort to alleviate this burden for informal caregivers and provide them with some professional help. Technology may be one solution. The World Health Organization supports this initiative, and finds it urgent to seek different ways of providing support to people who take care of individuals with dementia [13].

Technology is ubiquitous, and it penetrates all spheres of human life, including education, where information and communication technologies (ICT) are used in electronic education (i.e., e-learning), which is particularly used for distant education and as a support to traditional, face-to-face teaching for full-time students. E-learning is learner-centred to develop the student’s autonomy and independence while learning and make him/her responsible or his/her learning while providing him/her with needed skills and strategies for learning. Students can use it at any place and time and collaborate with other students. Learning has also become more interactive [14].

E-learning is primarily used as a component of traditional, face-to-face classes. This combined form of learning is called blended learning (BL), and it is widely used in healthcare in developed countries. L’Engle, Raney, & D’Adamo [15] claimed that e-learning enhanced provisions of health care and its services worldwide, and it enabled its delivery to remote regions and developing countries in particular.

Nine Lanterns [16] found that the use of e-learning in health care was primarily suitable for the training of future healthcare professionals in different regions and countries, and it was more cost-effective. The findings of their survey showed that 95% of subjects used online courses, and 80% used them as support for their face-to-face classes. Other research indicated the significance of the role of e-learning in knowledge retention, the understanding of particular health issues, continuous education, and the educating of future healthcare professionals [17–20]. Recent trends illustrate that e-learning courses also play important roles in inter-professional education and collaboration [21, 22]. E-learning courses may enhance inter-professional care in the sense of improved communication between healthcare staff and achieving desirable outcomes for people [23]. In case of informal caregivers, Official healthcare organizations, such as the Alzheimer’s Association, municipalities or healthcare staff, generally distribute e-learning programs [24]. These programs are mostly available for free or at reasonable cost, as in the case of iCare Stress Management e-Training Program, which focuses on reducing stress and depression of family caregivers [25].

Government and independent organizations also leverage e-learning to provide health information to the public of diseases awareness, such as HIV, and dietary and lifestyle advice [26]. The European Commission aims to fund projects that focus on improving digital health literacy [27].

The present article focused on the exploitation of e-learning as a support for informal caregivers of people with dementia and considered its benefits and limitations to provide proper and relevant care for this target group of people and maintain the quality of life of their caregivers.

## Methods

The topic of e-learning and its exploitation as a valuable support for caregivers of people with dementia was searched in three recognized databases, Web of Science, Scopus, and PubMed, from 2010 to 2018. The search period started in 2010 when e-learning began to appear widely [14]. Reference lists of the detected studies were checked to avoid omitting other important studies on the research topic. The authors used the following keywords: *e-learning* AND *caregivers*, *e-learning* AND *carers, e-learning* AND *dementia*, *e-learning* AND *dementia* AND *carers*, *e-learning* AND *dementia* AND *caregivers*. Two authors (BK, JM) detected 248 peer-review journal articles written in English. The largest share of these articles was identified in Scopus (91), followed by PubMed (82) and Web of Science (75). The titles and abstracts were thoroughly reviewed (61) and screened for duplication (16). Forty-five studies were screened, and 30 studies remained for full-text analyses.

The full-text articles were analysed and evaluated using the following inclusion and exclusion criteria. The following inclusion criteria were based on PICOS guidelines:

- The articles were published between January 1, 2010, and December 31, 2018.
- Only peer-reviewed journal articles written in English were included.
- Articles that involved older people with dementia and their informal caregivers were included.
- Only randomized controlled trials, experimental studies, or survey quality studies were included.
- The primary outcome concentrated on the use of e-learning as a support tool for informal caregivers.

The following exclusion criteria were used:

- Studies that focused on a different target group and disease were excluded, such as [13, 17–20, 23, 28–35].
- Descriptive studies that depicted the e-learning course for dementia caregivers, such as empirical studies [36–38], posters [39], or protocol trials [40].
- Review articles on the research topic [9, 41–44].

A backward search was also performed, i.e., references of detected studies were evaluated for relevant research studies that authors may have missed during their search, which identified another article. Therefore, six research articles were eventually analysed and evaluated.

**Fig. 1:**
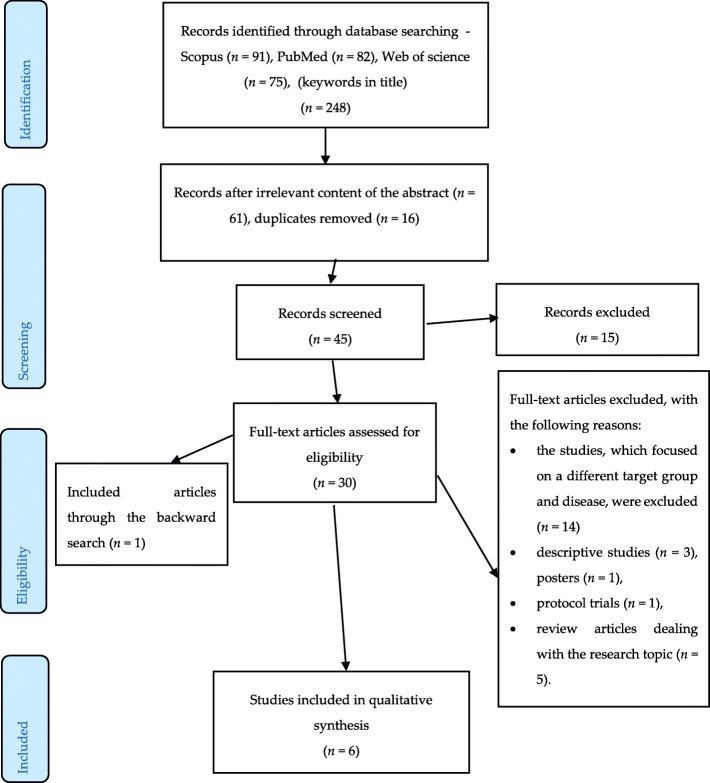
An overview of the selection procedure

Figure 1 below describes the selection procedure of the detected studies.

## Results

Six studies on the research topic were detected. Three studies were randomized controlled trials [24, 25, 45], two were survey quality studies [46, 48], and one was an experimental study [47]. The main topic in most studies concentrated on the use of e-learning in the improvement of psychological and emotional distress when caring for people with dementia, dissemination of knowledge about the disease itself and its management, enhancement of caregiver’s skills (social, behavioural, cognitive, or reflective), and the development a forum for their social network. E-learning support was primarily Internet based in the form of an educational training portal/program/course, and there was also a virtual simulation movie in one study [48]. These programs consisted of several modules or lessons and included different topics that focused on caregiver’s needs, such as *Cognitive Decline, Daily Tasks, Behavioral Changes, Social Activities,* or *You as a Caregiver*. For example, the virtual reality intervention Through the D’mentia Lens (TDL) enabled caregivers to see what dementia is like and how people with dementia feel [48].

Common methods were used to assess outcome measures, e.g., pre- and post-tests, online questionnaires, and statistical analyses. The detected articles lacked large subject samples. The number of participants was between 35 and 279 people. The period of assessment generally lasted from 3 weeks to 3–4 months without a follow-up period. Four studies [24, 45, 47, 48] were of multi-national origin (Australia, Denmark, the Netherlands, UK, Spain, and Poland), one study was a Chinese study [46], and one study was performed in USA [25]. The detected articles satisfied the basic quality criteria [49].

The results revealed that participants in all detected studies [24, 25, 45–48] were satisfied with the e-learning platforms, programs, and courses because the content helped them reduce their perceived stress and increase their empathy. The flexibility of these e-learning programs [24, 46] and their ease of accessibility from anywhere at any time [24, 25, 45–47], which meant less travelling, were other factors that motivated caregivers to participate in the intervention. Other benefits of e-learning support involved the personalized approach [45–47], the user-friendliness of e-learning programs [45–48], avoidance of stigmatized professional (psychiatric) help [46], development of a social network of informal caregivers [24], and the cost-effectiveness of the e-learning programs [25, 46]. In contrast, a high drop-out rate revealed the need to stimulate informal caregivers to participate in e-learning programs [25, 47]. One study [45] reported a reduction in self-reported sense of caring competence.

The findings of the detected studies are summarized in alphabetical order of their first author in Table 1 below.

**Table 1:** An overview of the findings of the selected studies

Study	Characteristics of subjects	Type of an e-learning tool and the length of the intervention	Outcome measures	Results, statistically significant differences
Hattink et al. [45] (RCT)	Participants, including informal caregivers (72/142, 50.7%), volunteers (24/142, 16.9%), and professional caregivers (46/142, 32.4%).	a multilingual e-learning portal - the European Skills Training and Reskilling (STAR); 2–4 months	questionnaires, statistical analysis	The STAR training portal seems to be useful, friendly, person-centred approach, increases caregiver’s empathy. In the experimental group, however, there was a significant reduction in self-reported sense of competence.
Ho et al. [46] (survey study)	279 dementia family carers.	dementia e-learning educational program (ADCarer.com); July 2011–January 2012 (survey)	self-administered questionnaire, statistical analysis	The results indicate that caregivers especially appreciated the convenience of the e-learning program, flexibility in choosing topics suitable to them, saving travelling time, and handling behavioural and psychological symptoms of dementia.
Kajiyama et al. [25] (RCT)	Informal caregivers - (*N* = 150) were randomly divided into the iCare Condition (ICC) or to the Education/Information-Only Condition (EOC).	iCare Stress Management e-Training Program; 3 months	self-report measures of stress (primary outcome), depression (CES-D) and quality of life (secondary outcomes), statistical analysis	The main outcome was a change in perceived stress; it was significant for the ICC but not the EOC (*p* = .017). Future efforts to improve dropout rate and increase caregiver’s motivation to participate in the program.
Nunez-Naveira et al. [24] (RCT)	61 informal carers (30 carers in the experimental group, 31 in the control group); age range: 25–88 years.	e-learning platform (understAID application) accessible through a smartphone or tablet; 3 months	the Center for Epidemiologic Studies Depression Scale (CES-D); self-completion questionnaire; statistical analysis	After using understAID the caregivers in the experimental group significantly decreased their depressive symptomatology; overall, 33.3% of the caregivers were satisfied with the application and approximately 50% of the participants assessed it as technically and pedagogically acceptable.
Pot et al. [47] (experimental study)	149 family caregivers (69.8% females, 30.2% males, average age: 61.5 years).	a guided self-help Internet intervention “mastery over dementia” (MoD)	Informant Questionnaire on Cognitive Decline in the Elderly, CES-D, Hospital Anxiety and Depression Scale, user evaluation, statistical analysis	MoD appears to be accessible for a broad range of family carers of people with dementia in terms of reach, adherence and user evaluation. The only drawback was a high percentage of carers who did not finish all lessons (55.7%).
Wijma et al. [48] (survey study)	35 informal caregivers (mean age = 55 years; 77% females, 23% males).	a virtual reality simulation movie and e-learning course: Through the D’mentia Lens (TDL); 3 weeks	pre- and post-test questionnaires, statistical analysis	TDL is feasible and acceptable for informal carers. Caregivers improved in empathy, confidence in caring for the person with dementia, and they had positive interactions with the person with dementia.

## Discussion

The findings of the detected studies showed that e-learning portals/programs/courses helped caregivers feel more confident about dementia care, enhanced their knowledge and skills, reduced their perceived stress, and enhanced their feelings of empathy, understandings and concern. These results are consistent with recent review studies on this topic [9, 41–44]. The results indicate that e-learning programs are feasible and acceptable for informal carers of people with dementia because these caregivers considered them useful, especially in improving their confidence about their care qualities [47, 48].

Kurz et al. [32] stated that the e-learning, especially web-based courses, was a new form of providing better healthcare learning, counselling, and assistance. Notably, learners studied according to their usual tempo. Therefore, this approach to learning enabled greater access to personalized learning and autonomy, which was reflected in the learners’ enhanced motivation [50]. Walsh [37] noted that e-learning courses should aim to meet the learner’s needs to be effective in providing relevant care for the target population groups. Nunez-Naveira et al. [24] also highlighted this goal in their study in which low scores were obtained for satisfaction and required modification to meet the informal caregivers’ needs in national, social, and cultural contexts.

The Chinese study [46] revealed that informal caregivers needed appropriate training because generally only younger caregivers and caregivers with a higher education level were willing to use the e-learning programs. Surr et al. [41] listed several key features that must be followed to achieve an effective training/educational program in dementia care. Some of these features may be applied in e-learning programs, such as tailored-made materials developed by a professional, the activities or tasks are realistic and based on experience, or use of different teaching methods. Moniz-Cook et al. [3] reported that without ongoing review of implementation of recommended action plans, e-learning interventions are not effective in reducing challenging behaviour in dementia compared to usual care.

There are several ongoing initiatives for e-learning programs, such as the RHAPSODY (Research to Assess Policies and Strategies for Dementia in the Young) program, which aims to provide informal caregivers with relevant skills and knowledge to help them overcome and deal with possible problems [32]. Moreno et al. [40] developed another project to reduce informal caregiver’s physical and mental burden and enhance their quality of life.

The demand for e-learning programs and resources is increasing because there is a need to guarantee an adequate quality of healthcare services and in this sense to provide professional and informal caregivers with relevant information and skills (cf. [34]). To assess the effectiveness of these e-learning programs, researchers should follow the model of Ruggeri, Farrington, & Brayne [35] for the assessment of e-learning courses in health care. The key performance indicators of this model are purpose, demographics of participants, acquired knowledge, resources, attitude and behaviour of the participants towards the e-learning program, their performance, time spent, quality assessment, audit, benefits, changes, and future use.

The limitations of this systematic review article are the lack of research studies on the research topic and the different methodologies, follow-up observations and assessment periods of the included studies. Surr et al. [41] also confirmed the small number of studies on family carers of people with dementia. Nevertheless, these inconsistencies may lead to an overestimation of the results on the exploitation of e-learning as a valuable support tool for caregivers of people with dementia, which may shed a negative light on the validity of the findings of these research studies (cf. [51, 52]).

## Conclusions

The findings of this study show that e-learning educational programs/courses help caregivers feel more confident about dementia care, reduce their stress and enhance their feelings of empathy, understanding and concern [53]. The results of this systematic review also show that the exploitation of e-learning as a support tool, especially for informal caregivers, in the management of dementia may be a promising method, but its implementation requires professional training of informal caregivers in the use of this technology.

## Data Availability

The search strategies used in this systematic review are included in the Methods, and all of the manuscripts informing this systematic review are listed in Table 1.

## Abbreviations

BL: blended learning
EOC: Education/Information-Only Condition
ICC: iCare Condition
ICT: Information and Communication Technologies
MoD: Mastery over Dementia
STAR: the European Skills Training and Reskilling
TDL: Through D’mentia Lens
